# The association between caffeine exposure during pregnancy and risk of gestational hypertension/preeclampsia: A meta‐analysis and systematical review

**DOI:** 10.1111/jog.15445

**Published:** 2022-09-25

**Authors:** Bangsheng Chen, Mengting Zhang, Yujing He, Yuexiu Si, Yetan Shi, Ke Jiang, Jingyi Shen, Jiaze Hong, Saisai Ni

**Affiliations:** ^1^ Emergency Medical Center, Ningbo Yinzhou No. 2 Hospital Ningbo Zhejiang China; ^2^ The Second Clinical Medical College Zhejiang Chinese Medical University Hangzhou Zhejiang China; ^3^ School of Basic Medical Sciences Zhejiang Chinese Medical University Hangzhou Zhejiang China

**Keywords:** caffeine, gestational hypertension, meta‐analysis, preeclampsia, risk

## Abstract

**Background:**

The potential effect of caffeine exposure during pregnancy on gestational hypertension (GH)/preeclampsia has attracted attention but remains unclear.

**Methods:**

A systematic literature search of PubMed, Embase, and Cochrane Library databases was performed until March 2022. Observational studies assessing the association between caffeine exposure during pregnancy and the risk of GH/preeclampsia were included. The study protocol was registered in PROSPERO: CRD42022322387.

**Results:**

Ten studies involving 114 984 pregnant women (2548 diagnosed with GH and 2473 diagnosed with preeclampsia) were included. Comparing caffeine exposure with noncaffeine exposure, no significant association was found between caffeine exposure during pregnancy and the risk of GH (odds ratio [OR] = 0.99, 95% confidence interval [CI]: 0.90–1.08, *p* = 0.800) and preeclampsia (OR = 1.13, 95% CI: 0.97–1.31, *p* = 0.114). Subgroup analyses comparing low to moderate doses with no/lowest doses showed that caffeine exposure during pregnancy was not significant associated with GH (OR = 1.00, *p* = 0.987) or preeclampsia (OR = 1.03, *p* = 0.648). Besides, subgroup analyses comparing high doses with no/lowest doses showed that caffeine exposure during pregnancy was not significant associated with GH (OR = 1.06, *p* = 0.623) or preeclampsia (OR = 1.18, *p* = 0.192).

**Conclusion:**

This study found that caffeine exposure during pregnancy was not significantly associated with the risk of GH/preeclampsia.

## Introduction

Hypertensive disorders of pregnancy (HDP) are one of the leading causes of maternal and perinatal morbidity and mortality worldwide, especially in low‐income and middle‐income countries.^1^ Gestational hypertension (GH) and preeclampsia are two types of HDP, accounting for 6%–7% and 2%–5% of diagnoses in pregnant women, respectively.^2^, ^3^ GH is defined as new‐onset hypertension with blood pressure ≥ 140/90 mmHg on two occasions at least 4 h apart after 20 weeks' gestation.^4^ Preeclampsia is defined as hypertension with proteinuria or other end‐organ dysfunction after 20 weeks of gestation.^5^ Furthermore, it is estimated that the latter complicates the pregnancy for about 3%–5% of the women who give birth.^5^ The etiology and predictors of GH/preeclampsia remain unclear.^6^, ^7^

Caffeine is the most widely consumed psychoactive substance worldwide.^8^, ^9^ Important dietary sources of caffeine include coffee, tea, caffeinated soda (cola), energy drinks, and other soft drinks.^10^, ^11^ Epidemiological evidence supports the benefits of caffeine for some chronic diseases, stating that moderate caffeine consumption is safe, but noting that excessive consumption can have negative effects on children and pregnant women.^12^, ^13^ WHO recommends that pregnant women consume no more than 300 mg caffeine per day.^14^ Additionally, a review of caffeine consumption recommends that pregnant women consume no more than two cups of coffee or four cups of tea per day.^12^

It is estimated that approximately 70% of women in the United States consume caffeine continuously during pregnancy,^15^ raising concerns about its potential effects on GH/preeclampsia. Nevertheless, observational studies on caffeine intake during pregnancy and the risk of GH/preeclampsia have yielded mixed results. Some papers displayed no relationship between caffeine intake during pregnancy and the risk of GH/preeclampsia.^16^, ^17^ Conversely, a nationwide birth cohort study by Kawanishi et al.^18^ reported that high dose of caffeine intake during pregnancy could increase the risk of GH/preeclampsia. Therefore, we conducted a meta‐analysis to discover the potential association between caffeine exposure during pregnancy and the risk of GH/preeclampsia.

## Methods

### Search strategy

We performed a systematic search on PubMed, Embase, and Cochrane Library databases from inception to 11 March 2022. The search items included (1,3,7‐trimethylxanthine OR caffeine OR tea OR coffee OR cola OR chocolate OR soft drink OR cocoa) AND (pregnant woman OR woman, pregnant OR pregnancy OR pregnance OR pregnancies OR maternity OR maternal OR maternally OR prenatal OR perinatal) AND (“pre‐eclampsia” [Mesh] OR “hypertension, pregnancy‐induced” [Mesh] OR “hypertension” [Mesh] OR pre‐eclampsia OR hypertension, pregnancy induced OR pregnancy‐induced hypertension OR induced hypertension, pregnancy OR gestational hypertension OR hypertension, gestational). Moreover, the references of the included articles were manually checked for additional sources. The entire review process was mapped using the Preferred Reporting Items for Systematic Reviews and Meta‐analyses (PRISMA) flow diagram and the study protocol was registered in PROSPERO: CRD42022322387.

### Eligible criteria

The meta‐analysis included studies with the following criteria, which^1^: were observational studies (cohort, cross‐sectional, or case–control studies)^2^; assigned participants consumed caffeinated beverages to the exposure group, and participants consumed no or the lowest dose of caffeinated beverages to the nonexposure group^3^; reported risk estimates for the association between caffeine exposure during pregnancy and the risk of GH/preeclampsia^4^; were published in English.

The exclusion criteria were as follows^1^: pregnant women with a medical history of hypertension and/or renal disease, history of HDP in previous pregnancies, and history of diabetes mellitus and gestational diabetes mellitus were included.^2^ The articles were designed to investigate the association between caffeine exposure before pregnancy and the risk of GH/preeclampsia.^3^ No exactable data was available.^4^ When more than one paper was published by the same author or institution, the paper with the highest quality was selected.

### Quality assessment and data extraction

Two researchers independently evaluated the eligibility of each study and exploited the desired information from it. Disagreements were arbitrated by a third researcher. The Newcastle–Ottawa Quality Assessment Scale (NOS) was used to assess the methodological quality of the included observational studies. Additionally, the following data were extracted: author, year of publication, country, study design, number of participants, time to recruit participants, pregnancy stage, outcome, caffeine intake sources, dietary assessment tool, and adjusted variables.

### Statistical analysis

All statistical analyses were performed using Stata software version 12.0, with *p* < 0.05 estimated as significant. OR and 95% CIs were applied as the effect sizes of caffeine exposure during pregnancy on the risk of GH/preeclampsia. As the dose and source of caffeine intake differed, a random effects model was used to combine effect sizes. Moreover, statistical heterogeneity among studies was assessed based on *I* ^2^ statistics. The values *I* ^2^ of 25%–50%, 50%–75%, and >75% were considered as low, moderate, and high heterogeneity, respectively.^19^ Subgroup analysis was established to identify sources of heterogeneity. When more than 10 items of statistics are included in the meta‐analysis, relevant calculation of publication bias and sensitivity analysis will be carried out.

## Results

### Characteristics of eligible research

**Figure 1:**
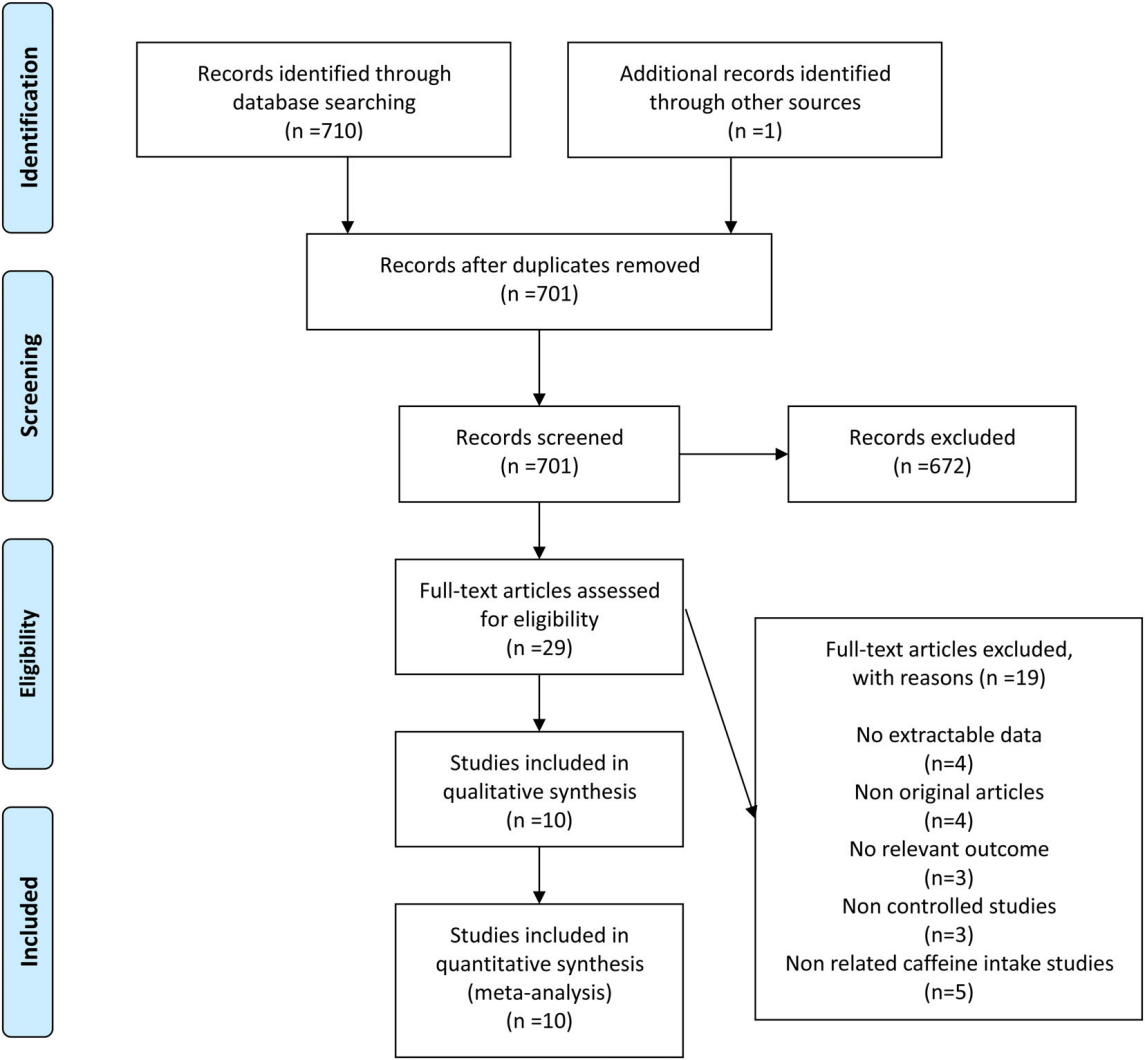
A schematic flow for the selection of articles included in this meta‐analysis

The flow chart of study selection is summarized in Figure 1. An initial search from various electronic databases found 701 relevant studies. After excluding unrelated papers, 10 studies eventually met the inclusion criteria.^16^, ^17^, ^18^, ^20^, ^21^, ^22^, ^23^, ^24^, ^25^, ^26^ The basic characteristics of selected studies are described in Table 1. One cross‐sectional study,^26^ two case–control studies,^21^, ^24^ and seven cohort studies^16^, ^17^, ^18^, ^20^, ^22^, ^23^, ^25^ were imported into meta‐analysis. All studies were published between 1997 and 2021, and these were conducted in Norway, USA, Canada, Netherlands, Ethiopia, Japan, and Brazil. A total of 114 984 pregnant women participated, including 2548 diagnosed with GH and 2473 diagnosed with preeclampsia. Dietary assessment of pregnant women was based on the food frequency questionnaire and interviews. In addition, caffeine sources for pregnant women included coffee, tea, chocolate, soft drinks, and energy drinks. Nine studies controlled the variables, except one that was not adjusted. Common variables adjusted for included studies were age, body mass index (BMI), education, and smoking.

**Table 1:** Characteristics of all the studies included in the meta‐analysis

Author	Year	Country	Study design	Time to recruit patients	Patients number		Outcome	Dietary assessment tool	Caffeine intake sources	Trimester considered	Comparisons		Adjustments
					Non exposure	Exposure					Non exposure	Exposure	
Wergeland	1997	Norway	Cross‐sectional	1989.10–1989.11	5292	280	Preeclampsia	Questionnaire	Coffee (cups/day)	Throughout pregnancy	≤4 cups/day	>4 cups/day	NA
Triche	2008	USA	Cohort	1996.9–2000.1	1681	63	Preeclampsia	Structured interview	Chocolate (servings/week)	First and third	<1 serving/week	1–4 servings/week	Age, BMI, education, smoking, parity, race, and clinic/private prenatal care provider.
												≥5 servings/week	
Wei	2009	Canada	Case–control	2003.1–2006.3	245	92	Preeclampsia	In‐person interview and structured questionnaire	Tea (cups/week)	Throughout pregnancy	No	Yes	Age, BMI, education, smoking, and abortion.
												0–3 cups/week	
												3–7 cups/week	
												≥7 cups/week	
									Coffee (cups/week)			yes	
												0–7 cups/week	
												≥7 cups/week	
									Soft drink (glasses/week)			yes	
												0–1 glasses/week	
												1–7 glasses/week	
												≥7 glasses/week	
Saftlas	2010	USA	Cohort	1988.4–1991.12	2325	57	Preeclampsia	Interview and FFQ	Chocolate (servings/week)	First and third	No	Yes	BMI and parity/abortion.
												<1 serving/week	
					2323	159	GH					1–3 servings/week	
												≥4 servings/week	
Bakker	2011	Netherlands	Cohort	2001–2005	7771	119	Preeclampsia	FFQ	Coffee and tea (units/day)	Throughout pregnancy	<2 units/day	2–3.9 units/day	Age, BMI, height, education, smoking, alcohol consumption, ethnicity, parity, stress in pregnancy, folate intake, and total daily energy intake.
												4–5.9 units/day	
												≥6 units/day	
					7653	237	GH						
Borgen	2012	Norway	Cohort	1999–2008	31 230	1703	Preeclampsia	FFQ	Sugar‐sweetened soft beverages, carbonated (ml/day)	First and second	No	8–30 mL/day	Age, BMI, height, education, smoking, leisure exercise, total energy intake, and dietary fiber.
												30–70 mL/day	
												≥70 mL/day	
Endeshaw	2015	Ethiopia	Case–control	2014.6–2014.9	302	151	Preeclampsia	In‐person interview and questionnaire	Coffee (cups/day)	Throughout pregnancy	No	Yes	Age, residence, MUAC value, fruit and vegetable intake, folate intake, coffee intake, and anemia.
Hinkle	2021	USA	Cohort	2009–2013	2719	83	Preeclampsia	FFQ	Coffee, tea, soda and energy drink (mg/day)	First and second	0 mg/day	1–100 mg/day	Age, BMI, education, race/ethnicity, marital status, and nulliparity.
												101–200 mg/day	
					2703	99	GH					>200 mg/day	
Kawanishi	2021	Japan	Cohort	2011.1–2014.3	56 300	1553	GH	FFQ	Coffee, tea (cups/day)	Throughout pregnancy	no	<1 cup/day	Age, BMI, education, smoking, alcohol consumption, study unit, parity, folate intake, and total caffeine intake.
												1–2 cups/day	
												≥2 cups/day	
Barbosa	2021	Brazil	Cohort	2010.1–2011.6	2750	425	GH	Structured questionnaire	Soft drink (times/week)	Second	<7 times/week	≥7 times/week	Age, education, and family income.

Abbreviations: BMI, body mass index; FFQ, food frequency questionnaire; GH, gestational hypertension; NA, not applicable.

### Quality assessment

The NOS consists of eight items and is divided into three dimensions, according to selectivity, comparability, and the study type‐outcome (cohort study) or exposure (case–control study).^27^ The NOS was performed to evaluate the quality of observational studies, and only high‐quality studies with an overall score ≥6 were included in the final analysis. Details of the quality assessment are displayed in Table S1, Supporting Information.

### Caffeine dose stratification

Since coffee/caffeine consumption was reported on various scales, we converted exposure data to a uniform measurement (mg/day). The amount of caffeine consumed per cup of coffee ranged from 74 to 107 mg, for tea 55 to 65 mg, for chocolate 16 to 56 mg and for soft drinks 24 to 95 mg.^28^, ^29^, ^30^ For dose subgroup analyses, study‐specific estimates of low to moderate dose caffeine intake included 1–3 servings/week,^22^ 2–3.9 and 4–5.9 units/day,^16^ 1–100 and 101–200 mg/day,^17^ 0–2 cups/day,^18^ 1–4 servings/week,^20^ 0–7 cups/week,^21^ 8–30 and 30–70 mL/day^23^ and high caffeine intake including ≥4 servings/week,^22^ >6 units/day,^16^ >200 mg/day,^17^ ≥2 cups/day,^18^ ≥7 times/week,^25^ >4 cups/day,^26^ ≥5 servings/week,^20^ ≥7 cups/week,^21^ and >70 mL/day.^23^

## Prognostic Analysis

### Gestational hypertension

**Figure 2:**
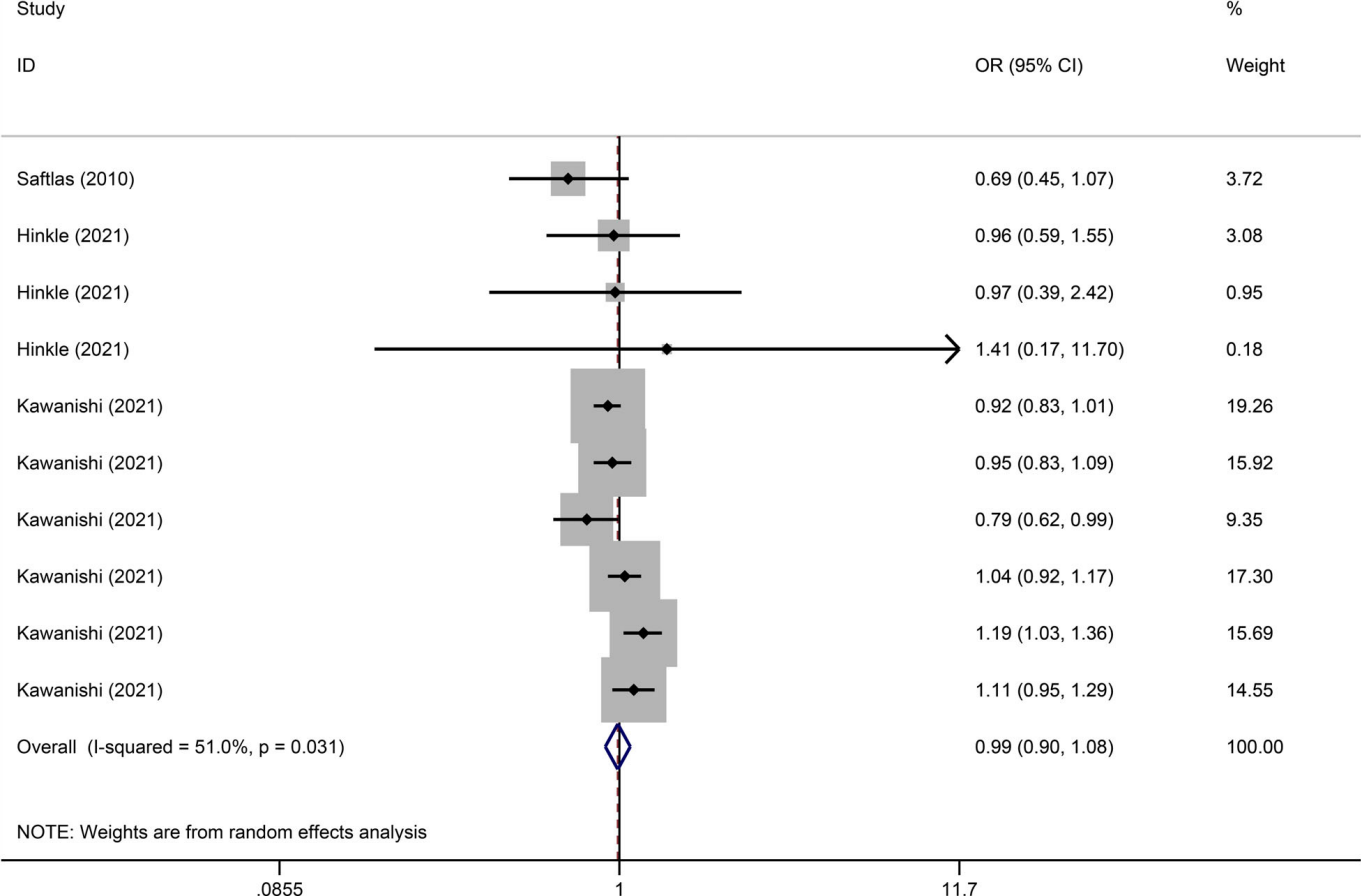
Forest plot of the association between caffeine exposure versus noncaffeine exposure during pregnancy and the risk of gestational hypertension (GH) (*p* = 0.800)

Three studies have been reported in GH comparing caffeine intake with no caffeine intake during pregnancy. The results revealed that caffeine intake during pregnancy was not significantly associated with the risk of GH (OR = 0.99, 95% CI: 0.90–1.08, *I* ^2^ = 51.0%, *p* = 0.800) (Figure 2).

Subgroup analysis of doses was performed to compare low to moderate dose with no/lowest dose and high dose with no/lowest dose, respectively. The results illustrated that the dose of caffeine consumption had no significant correlation with the incidence of GH (low/moderate dose: OR = 1.00, *p* = 0.987; high dose: OR = 1.06, *p* = 0.623, respectively) (Table 2).

**Table 2:** Subgroup analysis of the effects of caffeine intake on gestational hypertension and preeclampsia

	No. of studies	OR	95%CI	*p*	Heterogeneity (*I* ^2^) (%)
Gestational hypertension					
Low to moderate versus no/lowest	4	1.00	0.93–1.08	0.987	24.9
High versus no/lowest	5	1.06	0.84–1.33	0.623	64.7
Preeclampsia					
Yes versus no					
Coffee	2	1.78	1.15–2.74	**0.009**	0.0
Soft drink	2	1.10	1.00–1.21	0.056	14.2
Low to moderate versus no/lowest	6	1.03	0.91–1.17	0.648	15.0
Chocolate	2	0.63	0.36–1.09	0.098	0.0
Soft drink	2	1.07	0.95–1.20	0.280	9.6
High versus no/lowest	6	1.18	0.92–1.50	0.192	35.0
Coffee	2	1.24	0.70–2.19	0.456	65.8
Chocolate	2	0.66	0.37–1.17	0.153	0.0
Soft drink	2	1.40	0.79–2.47	0.246	48.9

*Note*: Bold value represents statistically significant differences (*p* < 0.05).

Abbreviations: CI, confidence interval; OR, odds ratio.

### Preeclampsia

**Figure 3:**
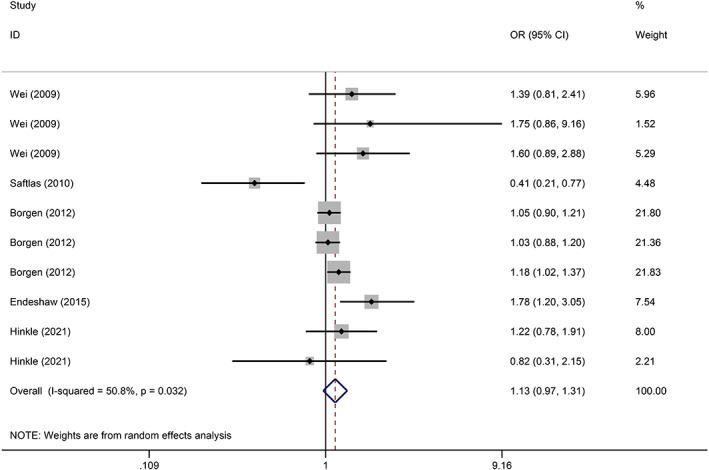
Forest plot of the association between caffeine exposure versus noncaffeine exposure during pregnancy and the risk of preeclampsia (*p* = 0.114)

Five studies referred to preeclampsia and compared caffeine intake with no caffeine intake during pregnancy. The pooled results depicted no significant association between caffeine exposure during pregnancy and the risk of preeclampsia (OR = 1.13, 95% CI: 0.97–1.31, *I* ^2^ = 50.8%, *p* = 0.114) (Figure 3). Besides, the sources of caffeine intake were analyzed by subgroup. The pooled results depicted that coffee (OR = 1.78, *p* = 0.009) and soft drink (OR = 1.10, *p* = 0.056) consumption during pregnancy was associated with an increased risk of preeclampsia, although the latter was not statistically significant (Table 2).

Subgroup analysis was performed on the doses and sources of caffeine intake. The pooled results of low to medium dose versus no/lowest dose showed no significant association between caffeine consumption during pregnancy and the risk of preeclampsia (OR = 1.03, *p* = 0.648). Moreover, pooled results of further subgroup analyses showed that consumption of chocolate (OR = 0.63, *p* = 0.098) and soft drinks (OR = 1.07, *p* = 0.280) during pregnancy was not significantly associated with the risk of preeclampsia (Table 2).

Similarly, the pooled results showed no significant correlation between caffeine consumption and the risk of preeclampsia (OR = 1.18, *p* = 0.192) at high doses versus no/lowest doses. Additionally, pooled results of further subgroup analyses showed that consumption of coffee (OR = 1.24, *p* = 0.456), chocolate (OR = 0.66, *p* = 0.153), and soft drinks (OR = 1.40, *p* = 0.246) during pregnancy was not significantly associated with the risk of preeclampsia (Table 2).

## Discussion

Our meta‐analysis aimed to investigate the association between caffeine exposure during pregnancy and the risk of GH/preeclampsia. The results demonstrated that caffeine exposure during pregnancy was not significantly associated with GH/preeclampsia. For preeclampsia, the results of subgroup analyses comparing caffeine exposure with noncaffeine exposure revealed that coffee and soft drink intake during pregnancy may increase the risk of preeclampsia, although there were only two studies in each subgroup analysis. Moreover, other subgroup analyses showed no association between caffeine intake during pregnancy and the risk of preeclampsia.

The relationship between caffeine consumption and HDP is of concern but remains controversial. In parallel to our findings, a meta‐analysis indicated that coffee consumption had no significant effect on hypertension.^31^ Furthermore, one review detected no clear connection between coffee exposure and the risk of hypertension.^32^ Nevertheless, dose–response meta‐analysis by Xie et al.^33^ and Grosso et al.^34^ both demonstrated that coffee consumption was inversely associated with risk of hypertension in a dose–response manner. Besides, a meta‐analysis proved a significant connection between soda consumption and the risk of hypertension.^35^ Additionally, a case‐crossover study of 286 women^36^ detected a strong inverse association of caffeine consumption with preeclampsia. Based on the above, the association between caffeine intake during pregnancy and GH/preeclampsia remains unclear.

These discrepancies can be explained by a number of underlying reasons. First of all, the limited number of studies covered is probably the main reason. Then, the respective effects of different sources of caffeine may interact, obscuring the actual correlation between caffeine exposure during pregnancy and the risk of GH/preeclampsia. In addition, the classification of caffeine dose levels varied between studies. Consequently, the lack of standardization in caffeine measurement cannot be ignored. Finally, guidelines from the American College of Obstetricians and Gynecologists (ACOG) recommended that pregnant women consume up to two cups of moderate‐strength coffee per day.^37^ Pregnant women have been shown to significantly reduce their caffeine intake, especially coffee during pregnancy.^38^, ^39^, ^40^, ^41^ Therefore, one possible explanation for our results is the low caffeine intake of pregnant women in the included studies, leading to a lower proportion of pregnant women consuming moderate to high doses.^16^, ^17^, ^42^

Although, as stated above, our results do not allow to draw a conclusion about causality, it is reasonable to speculate about possible mechanisms for the putative adverse effects of coffee or soft drink consumption on preeclampsia. Coffee contains several compounds such as kahweol, cafestol, chlorogenic acid, and trigonelline. These compounds have bidirectional effects on blood pressure regulation.^43^ It has been shown that caffeine has an acute blood pressure raising effect, especially in hypertensive individuals.^44^ In addition, caffeine and its metabolites belong to methylxanthines, which are nonselective adenosine receptor antagonists.^45^ Short‐term metabolic studies have shown that caffeine can sharply increase blood pressure by antagonizing adenosine A_1_ and A_2A_ receptors.^46^, ^47^, ^48^

The strengths of this article are that, to our knowledge, no meta‐analysis has examined the association between caffeine exposure during pregnancy and the risk of GH/preeclampsia. In addition, comprehensive subgroup analyses were established to explore the heterogeneity and compare the potential differences between different subgroups.

However, there are also some limitations. First, the number of articles included in this meta‐analysis was limited. Second, there was no standardization of caffeine in estimating caffeine consumption, which could lead to measurement bias. Third, all eligible articles were observational studies that had certain biases, such as recall bias and selection bias. Fourth, some studies did not control for covariates or ignored some potential residual confusions. Taking race as an example, although a large prospective cohort study has noted significant racial differences in HDP,^49^ only two studies in this meta‐analysis adjusted for race as a variable. Finally, the exclusion of non‐English articles may introduce publication bias.

The results of this meta‐analysis showed that caffeine exposure during pregnancy is not significantly associated with the risk of GH/preeclampsia, regardless of the dose or source of caffeine intake. Moreover, more appropriately designed, larger‐scale studies are needed to explore the impact of caffeine exposure during pregnancy on the risk of GH/preeclampsia.

## Author Contributions

Each author contributed significantly to concept and development of the present paper. Saisai Ni and Mengting Zhang designed the research process. Bangsheng Chen, Yujing He, and Yuexiu Si searched the database for corresponding articles and extracted useful information from the articles above. Yetan Shi and Ke Jiang used statistical software for analysis. Jingyi Shen and Jiaze Hong drafted the meta‐analysis. All authors had read and approved the manuscript and ensured that this was the case.

## Funding Information

This study was supported by the Natural Science Foundation of Ningbo, China (project No. 2018A610398), the Medical Health Science and Technology Project of Zhejiang Provincial Health Commission, China, (project No. 2022KY343), and the Science and Technology Program for Public wellbeing of Ningbo, China (project No. 2022AS069).

## Conflict of Interest

The authors declare there was no conflict of interest.

## Supporting information


**Table S1:** Quality assessment of included studies in this meta‐analysis.

## Data Availability

Data openly available in a public repository that issues datasets with DOIs. The data that support the findings of this study are openly available in PubMed at https://pubmed.ncbi.nlm.nih.gov/, Cochrane Library at https://www.cochranelibrary.com/, Embase at https://www.embase.com/, reference number 16‐18, 20‐26. The data that supports the findings of this study are available in the supplementary material of this article.
