# Supplementary material for: The association between caffeine exposure during pregnancy and risk of gestational hypertension/preeclampsia: A meta‐analysis and systematical review
Source: J Obstet Gynaecol Res. 2022 Sep 25;48(12):3045–55. doi: 10.1111/jog.15445 (PMC10087308; doi:10.1111/jog.15445)
Supplement: Supplementary file 1 — Table S1: Quality assessment of included studies in this meta‐analysis. [file JOG-48-3045-s001.docx]

**Supplementary Table 1. Quality assessment of studies included.**

| Author, year,  Study (Observational) | **Selection (Out of 4)** | | | | **Comparability**  **(Out of 2)** | **Outcomes(Out of 3)** | | | **Total**  **(Out of 9)** |
| --- | --- | --- | --- | --- | --- | --- | --- | --- | --- |
|  | Representativeness of exposed cohort | Selection of nonexposed cohort | Ascertainment  of exposure | Outcome not present at the start of the study |  | Assessment of outcomes | Length of follow-up | Adequacy of follow-up |  |
| Wergeland, 1997 | 1 | 1 | 1 | 1 | 0 | 1 | 0 | 1 | 6 |
| Triche, 2008 | 1 | 1 | 0 | 1 | 1 | 1 | 1 | 0 | 6 |
| Wei, 2009 | 1 | 1 | 0 | 1 | 2 | 1 | 1 | 0 | 7 |
| Saftlas, 2010 | 1 | 1 | 1 | 1 | 1 | 1 | 1 | 1 | 8 |
| Bakker, 2011 | 1 | 1 | 0 | 1 | 1 | 1 | 1 | 1 | 7 |
| Borgen, 2012 | 1 | 1 | 1 | 1 | 1 | 1 | 1 | 1 | 8 |
| Endeshaw, 2015 | 1 | 1 | 1 | 1 | 1 | 1 | 1 | 0 | 7 |
| Hinkle, 2021 | 1 | 1 | 1 | 1 | 1 | 1 | 1 | 1 | 8 |
| Kawanishi, 2021 | 1 | 1 | 1 | 1 | 2 | 1 | 0 | 1 | 8 |
| Barbosa, 2021 | 1 | 1 | 1 | 1 | 1 | 1 | 1 | 1 | 8 |

The observational studies were assessed by the Newcastle-Ottawa Quality Assessment Scale.
